# Stem Cells and Ion Channels

**DOI:** 10.1155/2013/238635

**Published:** 2013-05-16

**Authors:** Stefan Liebau, Alexander Kleger, Michael Levin, Shan Ping Yu

**Affiliations:** ^1^University of Ulm, 89081 Ulm, Germany; ^2^Tufts University, Medford, MA 02155-4243, USA; ^3^Emory University School of Medicine, Atlanta, GA 30322, USA

Once the zygote has formed by the fusion of the sperm cell with the oocyte, the early embryo quickly races through development, thereby gaining and losing several stages and types of stem cells. Only stem cells until the morula stage (~16–32 cell stage) are called totipotent which means the ability to form a complete, new organism. In contrast, the famous embryonic stem cell derived from the inner cell mass (embryoblast, ICM) exhibits pluripotency, resembling the differentiation capacity to form all cells of the organism, besides the extraembryonic structures when transplanted. These characteristics can also be found in the set-aside germline stem cells. Subsequently, after the formation of the three germ layers during gastrulation, germ layer stem cells are responsible for the generation of cells from the respective germ layer until gaining full functionality in the mature tissue network. The mature organism is full of various adult stem cells, also referred to as tissue-specific stem cells, which are mostly responsible for the cell turnover and regeneration in tissues.

Ion channels are represented by numerous families of pore forming structures which allow for the exchange of ions between the intra- and extracellular spaces or between the cytoplasm and organelles. These channel molecules vary, among others, in their ion specificity and conductance, their gating mechanisms, their subunit conformation, their downstream signaling including modulation of/by interacting molecules, or their expression profiles. Although ion channels have been thoroughly investigated in mature tissues and cells, their roles during development have remained elusive. Nevertheless, during recent years, it has become more and more evident that ion channels are involved in various cellular mechanisms during development, including germ layer generation, cell fate determination, migration, and stem cell differentiation. Moreover, the voltage gradients produced by several families of ion channels serve as instructive cues for positional information and organ identity during morphogenesis having been implicated in left-right asymmetry, craniofacial patterning, eye induction, and other roles in a range of model systems. Interestingly, not only the “simple” conductance of ions through biological membranes, but also the modulation of downstream signaling cascades linking bioelectrical events to changes in cell behavior is part of ion channel activity. Of note, many of these ion channel-mediated mechanisms are conserved throughout phylogenesis. 

To this end, we have selected a series of beautiful studies, which have investigated the role of ion channels within the development and stem cell differentiation. This includes not only relevant developmental model organism such as the *Xenopus laevis*, *Macrostomum lignano*, or the zebrafish, but also the whole spectrum of different stem cell populations being relevant to study differentiation.

D. Simanov and colleagues describe and discuss the role of ion channels during regeneration in the nonvertebrate flatworm *Macrostomum lignano*. They establish this animal as a versatile model organism for addressing these topics. They discuss biological and experimental properties of *M. lignano*, provide an overview of the recently developed experimental tools for this animal model, and demonstrate how manipulation of membrane potential influences regeneration in *M. lignano*. The authors show that modulation of ion channels in the worm greatly influences the regeneration of tissues and development of head-associated structures. These findings implicate that distinct ion channels and ion current adjustments are involved in the developmental events of invertebrates, making them an interesting model to study ion channel-mediated mechanisms throughout regeneration.

Going further in phylogenic evolution, V. P. Pai et al. provide evidence that ion channel-driven resting potential differences in key cell groups underlie consistent left-right patterning of neural tissues and organs in the developing *Xenopus larvae*. They show that the striking hyperpolarization of transmembrane potential demarcating eye induction usually occurs in the right eye field first. This asymmetry is randomized by perturbing pathways that regulate left-right patterning of the heart and visceral organs, suggesting that eye asymmetry is linked to mechanisms establishing primary laterality. Bilateral misexpression of a depolarizing channel mRNA affects primarily the right eye, revealing an additional functional asymmetry in the control of eye patterning. Further, they describe that the ATP-sensitive K^+^ channel subunit transcript, SUR1, asymmetrically expressed in the eye primordia is a good candidate for the observed physiological asymmetries. 

M. Keßler et al. provide an overview of the current knowledge from the zebrafish about ion channels and heart development. In that model, it has been observed that certain channel proteins such as scn5Laa and scn5Lab (representing the mammalian gene SCN5A), encoding a voltage-gated sodium channel, are not only present in the functional myocardium, but also are expressed in the developing heart during early embryonic stages. Indeed, the respective genes in mice or fish recapitulate these expression data as both in mammals and fish specific knock-down experiments lead to developmental defects of the heart. Interestingly, experiments indicate that the voltage-gated sodium channel exerts influence on cardiac development independent of ion flux. Furthermore, calcium channels which comprise different pore forming subunits are responsible for proper heart development as the pore-forming *α*1C subunit as well as the modulatory cytoplasmic *β*2.1 subunit specifically influences heart development in zebrafish. Finally, the authors discuss other ion channels and exchangers that are also involved in heart development such as the Sodium-Calcium-Exchanger, Ca^2+^-ATPase, Na^+^/K^+^-ATPase, and potassium channels.

The review of S. A. Becchetti Pillozzi et al. overviews the knowledge about the cell physiology of ion channels, especially of hematopoietic stem cells and one of their subpopulations, the mesenchymal stem cells (MSCs). It is already known that stem cells express a variety of ion channels, implicated in numerous cellular processes. This includes, for example, volume and resting potential oscillations accompanying the cell cycle. Other ion channel types control cell anchorage with the stromal matrix and cell migration as well as release of paracrine growth factors. Nevertheless, analyses of ion channel expression in MSCs seem to be highly varying. This may be an indication for several different populations in the cultures or cells at different cellular stages, respectively. 

M. Yamashita summarizes the mechanisms by which calcium ion fluctuations modulate the characteristics and behavior of neuroepithelial neural stem cells. In this respect, several channel proteins involving calcium channels, ligand-gated ion channels, and sodium and potassium channels mediate an ion equilibrium of the membrane and even the nuclear envelope. Calcium is thereby able to influence electric coupling of neighbored cells and is involved in the cell cycle by influencing DNA-synthesis during the S-phase.

The influence and role of large pore channels on neural stem cells (NSCs) and progenitor cells (NPCs) of the subventricular zone of the brain are summarized by L. E. Wicki-Stordeur and L. A. Swayne. They bring together knowledge about the interplay and individual functions of certain connexins, aquaporins, and pannexins in this cell system. Interestingly, these large pore channels do not only allow the passage of ions, but also mediate the highly controlled movement of small molecules. This, in turn, influences numerous cellular mechanisms such as adult neurogenesis. These pore-protein complexes, their regulation, and functions seem to be intimately connected and mediate cytoskeletal interactions, Ca^2+^ signaling, transcriptional regulation, ATP flux, and cell-cell communication between ventricular zone NSC/NPCs.

J. Aprea and F. Caligari summarize the current knowledge on the role of ion channels and pumps in the context of mammalian corticogenesis with particular emphasis on their contribution to the switch of neural stem cells from proliferation to differentiation and generation of more committed progenitors and neurons, whose lineage during brain development has been recently elucidated. They conclude that although several studies have, for example, depicted an influence of the membrane potential and ion currents on neural stem/progenitor cell proliferation, it is remaining elusive at what degree these *in vitro* findings may be translated to the *in vivo* situation.

The review article of M. Müller et al. sheds light on the various cardiac, developmental defects, which are mediated by the dysfunction or loss of distinct ion channels and finally lead to the distinct forms of long QT syndrome. During many years, numerous genetic defects have been investigated with the help of mouse models. Since the initial report on the generation of induced pluripotent stem cells that can be gathered from almost every individual, a human *in vitro* “model” has emerged. Therefore, this review highlights the respective stem-cell-based studies which investigate the underlying pathomechanisms and frequently show a predicted cellular behavior mimicking long QT syndrome.

Using human-induced pluripotent stem cells, L. Linta et al. compare a large number of ion channels and their subtypes which undergo differential expression starting from the initially used somatic cell further to the reprogrammed iPS cell and finally to the differentiated progeny, neurons and cardiomyocytes. This transcriptome array-based study depicts most of the commonly known ion channel families and subunits to draw a starting line for the study of ion channels in, for example, stem cells. Of note, numerous ion channel subtypes are highly upregulated after the process of reprogramming leaving iPS cells with a large number of expressed ion channels, at least on mRNA levels.

A. Illing et al. give insights into the differentially regulated expression patterns of an ion channel family known for its influence on stem cell differentiation towards the cardiac lineage. Here, they show the regulation of SK channel expression (small/intermediate conductance, calcium-activated potassium channels) during endodermal differentiation of human iPS cells. The authors not only provide a very robust protocol for the differentiation of human iPS cells into definitive endoderm cells, but also show that SK channels are highly regulated during this process.

 Together, these papers give an overview of an important emerging field. The synthesis of information on the genetics of individual channels with endogenous roles in cell regulation and the developmental biophysics that highlights the importance of resting potential for directing cell behavior, have exciting implications for basic biology and biomedicine of stem cell function.

*Stefan Liebau* *Stefan Liebau* *Alexander Kleger* *Alexander Kleger* *Michael Levin* *Michael Levin* *Shan Ping Yu* *Shan Ping Yu*

